# Skin Lesions Caused by HPV—A Comprehensive Review

**DOI:** 10.3390/biomedicines12092098

**Published:** 2024-09-13

**Authors:** Laura Maghiar, Mircea Sandor, Liliana Sachelarie, Ruxandra Bodog, Anca Huniadi

**Affiliations:** 1 Department of Surgical Disciplines, Faculty of Medicine and Pharmacy, University of Oradea, 1st December Square 10, 410073 Oradea, Romania; lauratodan@yahoo.ro (L.M.); bodogruxandra@gmail.com (R.B.); ancahuniadi@gmail.com (A.H.); 2 Preclinical Sciences Department, Faculty of Medicine and Pharmacy, University of Oradea, 1st December Square 10, 410073 Oradea, Romania; 3 Pelican Clinical Hospital Oradea, Str. Corneliu Coposu nr.14A-14B, 410450 Oradea, Romania; 4 Preclinical Sciences Department, Faculty of Medicine, Apollonia University, 700511 Iasi, Romania

**Keywords:** skin, vaccine, cancer, HPV

## Abstract

This narrative review provides a comprehensive analysis of skin lesions caused by human papillomavirus (HPV). Human papillomavirus is an infection involving a virus that is omnipresent and can range from benign wart lesions to malignant skin growths. This review includes an analysis of the skin manifestations caused by HPV, and the need for continued successful diagnostic techniques and treatment methods, given the increasing rates of infection among people worldwide. We reviewed all 135 studies related to pathophysiology involving skin, risk factors, and early detection methods like biopsy and molecular testing, from 2000 to 2023. The current treatments, including cryotherapy and laser therapy, are discussed, while the review emphasizes the role of HPV vaccination in preventing infection. Recommendations for the future would involve the improvement of public education and increased vaccine coverage, together with innovative therapies toward better management or control of skin diseases associated with the human papillomavirus (HPV). By advancing these recommendations, we will be in a better position to prevent and treat HPV skin conditions, thus improving the health condition of the general public across the world.

## 1. Introduction

Human papillomaviruses (HPVs) are DNA viruses infecting humans’ skin and mucous membranes [1]. These viruses are known for their ability to cause a variety of skin lesions, from common warts to precancerous and cancerous lesions [1,2]. Currently, there are more than 200 types of HPVs identified, many of which are associated with skin and mucosal lesions. HPV infections are among the most common viral infections in humans, affecting approximately 7–12% of the population at any given time [1]. According to Ishii et al., the complex interactions between the microorganisms that make up the microbiome and the immune system are of great importance in the prevention and treatment of respiratory infections [2].

HPV is a small, non-enveloped epitheliotropic virus with a circular genome of approximately 8000 bp. These viruses are classified into five genera based on DNA (Deoxyribonucleic acid) sequence homology in the L1 gene, which encodes the L1 capsid protein. Alpha protein includes HPV types that infect the skin and mucosal epithelium and is divided into low-risk and high-risk HPV. High-risk HPV, especially types 16 and 18, are well known for their critical role in the etiology of cervical cancer, supported by numerous epidemiological and experimental data. These types are also implicated in most other anogenital cancers and oropharyngeal squamous cell carcinoma [3,4,5].

Skin lesions caused by HPV are diverse and may include warts, flat warts, epidermodysplasia verruciformis (EV), and precancerous and cancerous lesions. Warts are the most common skin lesions associated with HPV and usually appear on the hands, feet, and other areas of the body. They are caused by HPV types 1, 2, 4, 27, and 57 [6]. Flat warts are smooth, skin-colored lesions that usually appear on the face, neck, hands, and wrists and are caused by HPV types 3 and 10 [7].

Epidermodysplasia verruciformis (EV) is a rare, hereditary condition characterized by extreme susceptibility to HPV infections. Patients with EV develop multiple warts on the body and are at an increased risk of malignant transformation. HPV types associated with EV include 5 and 8 [8,9]. This condition provides unique insight into how HPV infections can contribute to the development of skin cancer.

High-risk HPV, such as types 16 and 18, are associated with the development of precancerous and cancerous lesions. Although these lesions are more commonly associated with anogenital and oropharyngeal cancers, there is evidence to suggest that HPV may also play a role in cutaneous carcinogenesis [10]. For example, patients with epidermodysplasia verruciformis have an increased risk of developing cutaneous squamous cell carcinoma (cSCC) [11]. cSCC is the most common form of metastatic skin cancer and its incidence is increasing worldwide. cSCC is an advanced form of premalignant actinic keratosis (AK) that occurs in sun-exposed areas of the body [12]. Chronic exposure to ultraviolet (UV) radiation is known to be the main cause of cSCC development [13].

In recent years, it has become increasingly evident that HPV, in addition to chronic UV irradiation, immunosuppression, and genetic predispositions, is an important co-factor for the development of cSCC. HPV beta types are distributed across all five HPV genera, with the gamma genus being the most diverse and largest clade within the Papillomaviridae family [14]. The association between HPV beta types and the development of non-melanoma skin cancer (NMSC) was originally described in patients with epidermodysplasia verruciformis, an autosomal recessive predisposition in which patients develop cSCC mainly on sun-exposed areas of the body [15,16,17].

Molecular and functional studies of HPV beta E6 and E7 oncoproteins have demonstrated their negative effects on skin homeostasis. These proteins play a critical role in the viral life cycle by disrupting epithelial differentiation and immune homeostasis, promoting cell proliferation and expansion of the epithelial progenitor cell compartment to ensure viral DNA replication and progeny production. Yang et al. consider that the development of HPV-associated cancers is closely related to the role of oncogenes, especially the E6 and E7 proteins produced by HPV. Oncoproteins destabilize cellular defense mechanisms, such as p53 and Rb, and contribute to the malignant transformation of HPV-infected cells [18]. However, they also inhibit UV-induced DNA damage repair and apoptosis, thus enhancing the mutagenic capacity of UV exposure [19,20,21,22]. Understanding the mechanisms by which HPV contributes to skin carcinogenesis is essential for the development of effective prevention and treatment strategies. HPV vaccination and new immunotherapeutic approaches could prevent the development of HPV-associated skin tumors and improve the clinical management of these lesions [23,24,25,26,27]. The study by Roberts and Tinle shows the importance of vaccination and the global impact on the incidence of cervical cancer and other HPV-related conditions [28].

The development of skin cancer due to HPV infection is influenced by processes mainly related to the viral proteins E6 and E7. These proteins disrupt the function of skin cells. They promote cancer by interacting with different cellular communication pathways [29].

HPV’s E6 protein can degrade the p53 tumor suppressor protein by binding to E6AP, a ligase. This degradation occurs through the proteasome process, preventing p53 from carrying out its roles in DNA repair and programmed cell death. As a result, cells with mutations can grow uncontrollably [30].

Harden, M.E. and Munger highlight the role of high-risk HPV oncoprotein E6 in carcinogenesis; E6 interacts with the tumor suppressor protein p53, inhibiting its function and contributing to tumor cell survival and proliferation [31].

Attaching to the E7 protein deactivates the retinoblastoma protein (Rb), a tumor suppressor critical for regulating cell division by binding to Rb, E7 releases the transcription factor E2F, which leads to the activation of genes for the S phase of cell division. This leads to cell growth [32]. Recent research has shown that HPV infection can prevent the repair of DNA damage caused by exposure to ultraviolet (UV) light. The E6 and E7 proteins interfere with pathways that respond to DNA damage, such as nucleotide excision repair and non-homologous end joining (which increases the mutation rate in affected cells) [31].

In 2007, Narisawa Saito and Kiyono discussed that these changes could lead to skin cell layers and the formation of cellular carcinoma [33].

Recent studies have highlighted the role of persistent inflammation in shaping the environment around tumors, particularly in HPV infections. According to Wallace et al. in 2017, it is thought that this inflamed environment helps to evade the system and promote tumor growth [34,35].

## 2. Material and Method

A total of 135 articles published between 2000 and 2023 were identified through a comprehensive search in PubMed, Scopus, and other relevant databases using keywords such as “HPV”, “skin lesions”, “diagnosis”, and “treatment”. Of these, 105 articles were selected for inclusion in this review based on their focus on skin lesions caused by HPV and their contribution to understanding the diagnosis and treatment methods. Articles that did not specifically address skin lesions or lacked original data were excluded.

## 3. Epidemiology

Cutaneous warts affect between 7–12% of the population at any one time, with a higher prevalence among children and adolescents, with an estimated annual incidence of 10–20% in this age group [8]. According to the World Health Organization, approximately 5% of all cancers worldwide are caused by HPV infections, most of which are related to high-risk HPV types, especially 16 and 18 [9]. There are more than 200 different types of HPV, of which about 40 can infect the anogenital region and the oral cavity. HPV infections are usually asymptomatic and transient, but certain high-risk types of HPV can lead to the development of cancers, including cervical, anal, penile, vaginal, vulvar, and oropharyngeal cancer. HPV is extremely common, and most sexually active people will contract at least one type of HPV during their lifetime. An estimated 79 million Americans are infected with HPV, with approximately 14 million new infections occurring each year in the United States. The global prevalence of HPV varies significantly by region, age, and sexual behaviors [20,21,29].

HPV is transmitted through direct contact with infected skin or mucous membranes. The main way of transmission is vaginal, anal, or oral sexual contact. The infection can also be passed from mother to child during birth, resulting in recurrent respiratory papillomatosis.

### Types of HPV

HPV is divided into two main categories: low-risk types and high-risk types (Table 1). We take into account only the most relevant HPV types for the study.

Low-risk HPV types, such as HPV 6 and 11, are linked to genital warts and respiratory papillomas. High-risk types, such as HPV 16 and 18, are associated with anogenital and oropharyngeal cancers. HPV 16 is particularly oncogenic and responsible for most cases of cervical cancer [37,38,39,40,41]. Vaccination with Gardasil and Cervarix is the most effective method of preventing HPV-related infections and diseases. Regular Pap smear screening and HPV DNA testing are essential for early detection of precancerous lesions [42,43,44,45,46,47].

The epidemiology of HPV infection emphasizes the need for preventive measures such as vaccination and screening. Understanding the distribution and risk factors of HPV is essential for the development of effective strategies to reduce the incidence and mortality of HPV-associated cancers [26].

## 4. Pathogenesis and Mechanisms of HPV Infection

According to Humans I.W.G., HPV infects epithelial cells through skin or mucous membrane micro-abrasions [48]. The virus accesses the basal layer of the epidermis, where it replicates and induces cell proliferation, leading to the formation of warts (Table 2). In persistent infections, HPV DNA can be integrated into the host genome, leading to the malignant transformation of cells [10].

In some cases, infected cells can undergo malignant transformation, leading to the development of cancer, such as cervical cancer [35].

## 5. Diagnosis of Skin Lesions Caused by HPV

The appearance of the lesions is important and induces the type of clinical diagnosis. Tests such as PCR (polymerization chain reaction) can detect the presence of HPV.

PCR is a method that amplifies the specific sequences of viral DNA, even if they are in very small amounts, and thus ensures a precise detection of different types of HPV. PCR may be combined with other techniques to confirm the type of HPV involved in the skin lesion and to determine the viral load, like sequencing or in situ hybridization. Gender et al. consider that biopsy is indicated if the lesions are suspicious or atypical [41].

In the view of Smith, J., the initial diagnostic step entails inspecting the skin for HPV-related lesions [42]. This visual assessment helps identify warts, flat warts, and suspicious lesions that may need exploration. Brown et al. mentioned that dermoscopy can complement examination by offering an up look at the skin details not visible to the naked eye [43].

According to White and Green, when there is uncertainty in diagnosis or if lesions seem unusual, a skin biopsy becomes necessary. This procedure involves obtaining a tissue sample for analysis to confirm HPV presence and distinguish between malignant lesions [44].

As per Wright’s perspective, HPV DNA testing plays a role in identifying the HPV type involved [44]. This molecular technique detects HPV DNA within skin lesions, with sensitivity and specificity levels [46].

Recent developments have transformed HPV DNA testing into a resource, for detecting and handling diseases associated with HPV at a stage [47].

These diagnostic methods complement each other and provide a comprehensive approach to the diagnosis of HPV-related skin lesions, Table 3.

### 5.1. News in HPV Diagnosis

#### 5.1.1. HPV DNA Testing

WHO has prescribed the utilization of HPV DNA testing as the favored strategy of screening for cervical cancer. This test identifies the high-risk strains of HPV that are dependable for nearly all cases of cervical cancer. HPV DNA testing is more exact and objective than conventional strategies such as cytology (Pap test) and visual review with acidic corrosive [47,48,49,50,51,52].

#### 5.1.2. Self-Sampling of Tests

WHO moreover prescribes the utilization of self-sampling for HPV DNA testing. Ponders appear that ladies feel more comfortable taking their claim tests at domestic, which can increment the screening rate and contribute to the worldwide objective of testing 70% of ladies by 2030 [53,54].

**Table 3:** Diagnosis of skin lesions caused by HPV.

Diagnostic Method	Description
Clinical examination	Visual assessment of skin lesions [49,50,51,52]
Dermoscopy	Using a dermatoscope to examine skin lesions in detail [53]
Skin biopsy	Taking a tissue sample for histopathological examination [55]
HPV DNA testing	Detection of HPV DNA in skin lesions is a very sensitive and specific method for HPV typing [41]

#### 5.1.3. Oral HPV

Most oral HPV diseases are cleared by the safe framework in less than a year and a half. Be that as it may, a small rate of individuals holds the contamination long-term, which increases the hazard of creating oropharyngeal cancers [49,50,51,52].

## 6. Treatment of Skin Lesions Caused by HPV

Treatment of skin lesions caused by HPV depends on the type, location, and severity of the lesion and the patient’s response to previous treatments [53,54]. The main treatment options include physical therapy, chemotherapy, and immunotherapy (Table 4). The goal of treatment is to eliminate the lesion, relieve symptoms, and prevent recurrence.

The way HPV-related skin lesions are treated depends on factors such as the type, location, and severity of the lesions and how the patient has responded to previous treatments. Treatment options include therapies such as cryotherapy and laser therapy, as well as chemical treatments and immunotherapy.

As described by Parker and Davis, cryotherapy involves freezing the lesions with nitrogen, which is a simple and effective way to remove warts [47]. However, several sessions may be needed but the procedure can be uncomfortable [55,56]. Electrocautery, says Bailey, uses heat to remove tissue but can be painful and often requires a local anesthetic [50].

For extensive lesions, Foster suggests laser therapy as a targeted approach to vaporize the affected tissue [51]. While effective, this method can be costly. It requires equipment.

In cases where other treatments prove ineffective, surgical excision may be needed for resistant lesions; surgery is effective but can cause scarring and usually requires anesthesia [58].

Butler and Scott suggest that aside from therapies, using treatments like salicylic acid and cantharidin topically is an option for treating warts. These methods are generally user-friendly but may irritate skin and need to be applied regularly [53].

Evans points out that immunotherapy is an approach for dealing with HPV-related skin issues, especially for patients with recurring or challenging lesions [60]. Vaccines and other treatments that modulate the system aim to enhance the body’s response to HPV offering a long-term solution.

Peterson believes that by combining these treatment choices, along with measures like vaccination and regular screening, there is a potential to reduce the occurrence and severity of HPV-related skin problems leading to better outcomes for patients [55].

Recent progress, in treating HPV has been centered on developing therapies and exploring research paths to enhance the management of HPV-related issues like skin lesions.

According to Frazer, one area of focus is vaccines meant for individuals already infected with the virus [56]. These vaccines trigger the system to detect and eliminate cells infected with HPV using delivery methods such as vectors or DNA. The ability to provoke a response against HPV-infected cells is proving effective in trials [54,55].

Another innovative strategy, from Kennedy et al., involves utilizing the CRISPR Cas9 gene, a gene-editing technology that involves two essential components: a guide RNA to match a desired target gene, and Cas9—an endonuclease that causes a double-stranded DNA break, allowing modifications to the HPV genome within cells. By editing DNA, CRISPR Cas9 can effectively deactivate the virus, potentially preventing cancer progression. Initial studies indicate that CRISPR Cas9 can pinpoint and disrupt cancer-linked HPV16 and HPV18 genomes [56,57].

Cohen et al. considered that immunotherapies like pembrolizumab and other checkpoint inhibitors hold promise in treating HPV-related cancers by enhancing the capacity of the system to attack tumor cells carrying HPV [59].

Researchers are presently studying the advantages of combining these inhibitors with treatments such as radiation or chemotherapy to enhance treatment outcomes [53].

Bommareddy et al. introduced oncolytic virotherapy, which represents another innovative strategy and is genetically engineered to selectively infect and kill cancer cells while sparing normal tissues. In the realm of cancer treatment, oncolytic virotherapy stands out as a groundbreaking method that harnesses engineered viruses to specifically invade and eradicate cells. These specialized viruses are engineered to home in on tumor cells multiplying within them and leading to their disintegration. This dual action does not eliminate cancer cells directly. Also, it triggers a heightened immune response in the body enabling it to better identify and combat cancer cells [60].

Studies on compound therapy are also progressing, with plant-based compounds that exhibit promise in combating HPV. Substances such as curcumin, green tea catechins, and resveratrol have shown potential in fighting HPV based on studies [61]. These compounds could potentially hinder HPV replication and trigger cell apoptosis in infected cells, boosting responses. However, more research is required to determine their effectiveness, in real-world scenarios.

Enhancements in nanotechnology have led to the creation of delivery systems aimed at enhancing the efficiency of treatments for HPV. By customizing nanoparticles, it becomes possible to transport substances to cells infected with HPV, thereby boosting drug concentration at the infection site and reducing general side effects. As an illustration, scientists are currently exploring nanoparticles designed to administer siRNA for suppressing the activity of HPV oncogenes potentially lowering levels and thwarting cancer progression [62,63].

Epigenetic treatments are becoming a strategy for addressing HPV-related cancers [63]. The goal of these therapies is to undo the effects of HPV that silence genes responsible for suppressing tumors and encourage cancer development. Medications tailored to address these changes are currently undergoing trials and displaying promise in restoring cell functions and halting tumor advancement. As research advances and additional trial results emerge, there is a potential for transforming the treatment landscape of HPV, offering avenues for preventing and managing diseases associated with the virus [63]. These technologies could significantly change the landscape of HPV treatment and offer new ways to prevent and treat HPV-related conditions as they continue to evolve and clinical trials provide more data.

### 6.1. Prevention of Skin Lesions Caused by the HPV Virus

HPV infection can be advantageous to a diversity of skin lesions, from common warts to precancerous and cancerous lesions. In the context of the increasing incidence of these infections, prevention becomes indispensable to reduce the long-term impact of HPV on public health [50,52,55,64].

The HPV vaccine has demonstrated strong effectiveness in preventing high-risk HPV strain infections that are linked to the formation of skin lesions and various cancers in the anogenital and oropharyngeal regions [64,65,66,67,68,69,70,71]. HPV infection can lead to a multitude of skin lesions, from common to precancerous and cancerous lesions [72,73,74,75,76]. Prevention of high-risk HPV infections, which are associated with the formation of skin lesions and various types of cancer in the anogenital and oropharyngeal regions, is effectively achieved by available HPV vaccines such as Gardasil and Cervarix [77,78]. Gaspari points out that although there are effective vaccines against high-risk HPV types, there are no vaccines available for HPV types that cause benign skin lesions [4]. Vaccines like Gardasil and Cervarix are accessible and offer protection against the main oncogenic types of HPV, such as HPV 16 and 18, that cause the majority of cervical cancer cases. Gardasil 9 broadens this protection to encompass additional high-risk strains (Table 5).

It is advised that both girls and boys receive vaccinations between the ages of 9–12, before becoming sexually active, to achieve optimal protection. Research has indicated that receiving the HPV vaccine at a young age offers a better and more enduring defense against HPV infections and related conditions.

Health education is essential in preventing HPV infections by promoting higher vaccination rates and encouraging regular screening. The consistent and correct use of condoms can significantly lower the risk of HPV transmission [72,73,74].

Routine screenings for cervical cancer, such as Pap smears and HPV DNA tests, are crucial for the early identification of precancerous conditions and early-stage cancers (Table 6). Papanicolau smears help in detecting cellular changes that may lead to cancer, while HPV DNA tests can identify high-risk HPV types (WHO, 2017).

Other preventive strategies include quitting smoking, as it contributes to the persistence of HPV infections and the progression of precancerous lesions [10]. Leading a balanced lifestyle with a nutritious diet and regular exercise is also vital for maintaining a robust immune system.

Table 6 highlights the strategies to prevent HPV infections and skin lesions, providing an overview of the different approaches used. The different preventive approaches for HPV infections and skin lesions are presented, highlighting the methods, target populations, benefits, disadvantages, and challenges associated with each strategy. It is a comparison of various preventive measures such as vaccination, public education programs, regular screening, and lifestyle modifications. For every measure, we separately showed its specific objectives (e.g., reducing HPV transmission), and the population it targets (e.g., adolescents, sexually active individuals).

These challenges require a multi-faceted strategy that involves improving accessibility, education, and resources, as well as adapting programs to the cultural and religious needs of the targeted communities [69]. Collaboration between international organizations and local communities is essential to advance HPV prevention and reduce the incidence of associated cancers [70].

Public health campaigns and education programs are essential in the fight against HPV. As part of a comprehensive public health strategy, public health campaigns help to ensure that more people are informed, protected, and empowered to take control of their health.

To lower the spread of human papillomavirus (HPV) and combat the range of health issues linked to the virus such as skin abnormalities, anogenital cancers, and oropharyngeal cancers, public health campaigns and educational programs are crucial. These efforts form a key component of public health tactics designed to manage HPV transmission and minimize its effects on society.

Public health promotion is very important in educating people about HPV, how it is spread, and the potential health risks it poses. Frazer showed that many people are unaware of the prevalence of HPV and its links to serious health problems such as cancer [56]. Public health initiatives stress the importance of vaccination, especially for young people before they become sexually active. These efforts typically aim to reach out to parents, healthcare providers, and young adults to encourage them to prioritize vaccination [99,100,101,102].

For HPV-related diseases, such as cervical cancer, regular screening is very important. The importance of Pap tests is given by educational programs and HPV DNA tests, which are tools for the early detection of precancerous lesions and early-stage cancers. These campaigns, by promoting routine screening, help detect HPV-related disease in its earliest and treatable stages [52].

Ferris et al. considered that the health campaigns work to reduce the stigma associated with sexually transmitted infections, including HPV, by normalizing discussions about sexual health and emphasizing that HPV is a common infection that can affect anyone who is sexually active [58].

Education programs often include information on safe sexual practices, such as consistent and correct condom use, which can reduce the risk of HPV transmission. However, condoms do not provide complete protection against HPV but can significantly reduce the risk [63].

There are health campaigns specifically designed to reach populations at higher risk of HPV infection and related diseases, such as men who have sex with men (MSM), people with multiple sex partners, and people with compromised immune systems [60].

Modern technology like digital platforms, social media, and mobile technology seems to be a modern way for health campaigns to raise awareness of HPV.

### 6.2. Management of Skin Lesions Caused by HPV

Management of skin lesions caused by HPV lesions varies depending on the type of lesion, its location, and the patient’s response to previous treatments [80,81,82,83,84]. Management of skin lesions caused by HPV requires a multifaceted approach that includes physical, chemical, and immunotherapies. Vaccination remains the most effective method of prevention. Appropriate treatment, together with preventive measures, can significantly reduce the incidence and severity of HPV skin lesions and improve the quality of life of affected patients [85,86,87].

## 7. Conclusions

Skin lesions caused by HPV represent a serious health problem and have a significant impact on a patient’s quality of life. Treatment of skin lesions and prevention of HPV infection requires a combination of accurate diagnosis, effective treatment, and preventive measures. Continued health education, treatment innovations, and widespread access to vaccination are critical to long-term success in the fight against HPV and its complications. Early diagnosis, effective treatment, and vaccination prevention are essential for treating skin lesions caused by HPV; however, challenges like limited access to health services, educational barriers, low public awareness, and cultural and religious resistance, as well as funding and logistical issues, make it difficult to address.

## Figures and Tables

**Figure 1:**
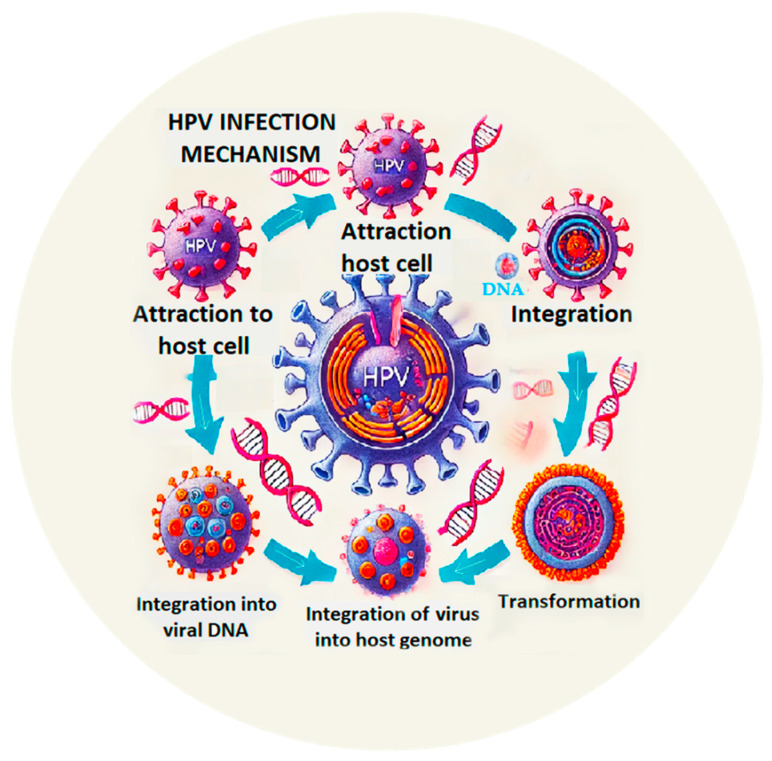
HPV infection mechanism.

**Table 1:** Types of HPV.

Types of HPV *	Risk	Associated Diseases	Observations
HPV 1, 2, 4	Low	Skin warts	Commonly causes plantar warts and verruca vulgaris [36]
HPV 6, 11	Low	Genital warts, recurrent respiratory papillomatosis	Gardasil and Gardasil 9 vaccines protect against these types [21]
HPV 16, 18	High	Cervical cancer, anal cancer, oropharyngeal cancer, penile cancer, vaginal cancer, vulvar cancer	HPV 16 is responsible for most cases of cervical cancer [27]
HPV 31, 33, 45	High	Precancerous lesions of the cervix, other anogenital cancers	Partial protection by vaccination (Gardasil 9) [25]
HPV 5, 8	High	*Epidermodysplasia verruciformis*, squamous cell carcinoma of the skin	Rare, associated with a rare genetic condition [8,9]

* The most relevant HPV types for the study.

**Table 2:** Mechanism of HPV infection.

Mechanism of Infection (Figure 1)	Description
Attachment of the virus to the cell surface	HPV attaches to specific receptors on the surface of epithelial cells through the L1 protein in its capsid [11]
Entry of the virus into the cell	The viral DNA is released which is then transported into the nucleus of the host cell.
Integration	HPV DNA integrates into the host cell’s genome. For high-risk HPV types, such as HPV 16 și 18, this integration can disrupt host genes and initiate carcinogenesis [12]
Viral shedding and transport	The expression of viral genes E6 and E7 inactivates the tumor suppressor proteins p53 and Rb, leading to uncontrolled cell proliferation and the development of precancerous lesions [13]

**Table 4:** Treatment of skin lesions caused by HPV.

Treatment	Description	Advantages	Disadvantages
Cryotherapy	Freezing lesions with liquid nitrogen [55]	Simple, fast, and effective method	Discomfort and requires multiple sessions
Electrocautery	Using electrically generated heat to destroy infected tissue	Allows precise removal of lesions [56]	Painful and may require local anesthesia
Laser therapy	Using a laser to vaporize the lesions [57]	Effective for multiple or recurring injuries	Expensive and requires specialized equipment
Surgery	Surgical excision of the lesions [58]	Effective for large lesions or resistant to other treatments	It requires anesthesia and can leave scars
Salicylic acid	A keratolytic that helps dissolve keratin [59]	Available without a prescription and easy to use	May irritate healthy skin and requires regular applications
Cantharidin	The beetle extract is used to treat warts by inducing blister formation [60]	Painless and effective application for children	It requires medical supervision and may cause pain
Podophyllin and podophyllotoxin	Cytotoxic agents that inhibit cell division in HPV lesions [51]	Effective for anogenital warts	They can be irritating and require a medical application
Therapeutic and experimental vaccines	Designed to boost the immune system to fight existing HPV infections [55]	Provide a long-term solution for recurring infections	Experimental and not yet widely available

**Table 5:** HPV vaccination process.

Stage	Description	References
Administration of HPV vaccine	Intramuscular injection in the deltoid muscle of the arm	[17,19,20,23]
Presentation of antigen	Phagocytosis by dendritic cells, which uptake the HPV antigen from the vaccine	[17,23,24,25]
Activation of T cells	Antigen presentation on MHC Class II molecules by dendritic cells	[17,23,24,25]
Stimulation of B cells	Activated T cells stimulate B cells to differentiate into plasma cells	[17,23,24,25]
Production of antibodies	Plasma cells produce specific antibodies against HPV	[17,23,24,25]
Immune Memory	Formation of memory B and T cells to provide long-term protection against HPV infection	[17,23,24,25]

**Table 6:** Preventive approaches for HIV infections.

Approach	Description	Prevention	Population	Advantages	Disadvantages	Challenge
Vaccination	Administering HPV vaccines to prevent infection [62]	Stimulates immune response to prevent HPV infection	Pre-adolescents, adolescents, and young adults	Highly effective in preventing HPV-related cancers and warts	Limited access in some regions	Limited access in some regions [75,76,77,78]
Vaccine availability	Ensuring widespread distribution and availability of HPV vaccines [17,19,20]	Increases vaccine uptake and herd immunity	General population	Reduces the prevalence of HPV-related diseases	High costs and logistical challenges	High costs [68,69,70,71,72,73,74,75,76,77,78,79,80,81]
Public education programs	Informing the public about HPV and prevention methods	Raises awareness and promotes preventive behaviors	General population	Increases knowledge and reduces stigma	Cultural and religious acceptance issues	Cultural and religious acceptance [76,77,78,79,80,81,82,83,84,85,86,87,88,89,90]
Screening	Regular testing to detect HPV and related lesions early [24,25]	Early detection and treatment of precancerous changes	Sexually active individuals, women aged 21–65	Reduces the risk of progression to cancer	Reduced accessibility to screening tests	Reduced accessibility to screening tests [91,92]
Regular Pap tests	Cytological examination to detect cervical abnormalities [23,24]	Identifies precancerous changes and early-stage cancer	Women aged 21–65	Effective in early detection of cervical cancer	Lack of public information and awareness	Lack of public information [93,94,95,96]
HPV DNA tests	Molecular testing to detect high-risk HPV strains [23,24,25]	Identifies high-risk HPV infections that may lead to cancer	Women aged 30 and older	More accurate than Pap tests in detecting high-risk HPV	Limited costs and infrastructure	Limited costs and infrastructure [97,98,99]
Education and awareness	Programs to educate about HPV, its risks, and preventive measures [12,15,26,27,28]	Dispels myths and promotes vaccination and screening	General population	Reduces misinformation and increases preventive behaviors	Misinformation and cultural resistance	Misinformation and myths [100,101]

## References

[1] Majewski S., Jablonska S. (2002). Epidermodysplasia verruciformis as a model of human papillomavirus-induced genetic cancer of the skin. Arch. Dermatol..

[2] Ishii K., Suzuki H., Nagai M., Ohno S. (2020). The Role of the Microbiome in the Immune Modulation of Respiratory Tract Infections. Front. Microbiol..

[3] Antonsson A., Karanfilovska S., Lindqvist P.G., Hansson B.G. (2003). General acquisition of human papillomavirus infections of the skin occurs in early infancy. J. Clin. Microbiol..

[4] Gaspari A.A. (2003). Human papillomaviruses and skin cancer. Clin. Dermatol..

[5] Aldabagh B., Angeles J.G., Cardones A.R., Arron S.T. (2013). Cutaneous squamous cell carcinoma and human papillomavirus: Is there an association?. Dermatol. Surg..

[6] Arora R., Jain A. (2017). Human papillomavirus infection in human immunodeficiency virus-positive patients: The changing face of head and neck cancer. Indian J. Sex. Transm. Dis..

[7] Ciotti M. (2020). The role of human papillomavirus in HIV progression. Curr. HIV Res..

[8] Clifford G.M., Franceschi S. (2009). Human papillomavirus types among women infected with HIV: A meta-analysis. Aids.

[9] Kawana K., Yasugi T., Taketani Y. (2009). Human papillomavirus vaccines: Current issues & future. Indian J. Med. Res..

[10] Gillison M.L., Chaturvedi A.K., Lowy D.R. (2008). HPV prophylactic vaccines and the potential prevention of noncervical cancers in both men and women. Cancer.

[11] Doorbar J. (2005). The papillomavirus life cycle. J. Clin. Virol..

[12] Stanley M. (2010). Pathology and epidemiology of HPV infection in females. Gynecol. Oncol..

[13] Chaturvedi A.K., Engels E.A., Pfeiffer R.M., Hernandez B.Y., Xiao W., Kim E., Jiang B., Goodman M.T., Sibug-Saber M., Cozen W. (2011). Human papillomavirus and rising oropharyngeal cancer incidence in the United States. J. Clin. Oncol..

[14] Munoz N., Castellsague X., de Gonzalez A.B., Gissmann L. (2006). Chapter 1: HPV in the etiology of human cancer. Vaccine.

[15] Zur Hausen H. (2009). Papillomaviruses in the causation of human cancers—A brief historical account. Virology.

[16] Khan M.J., Castle P.E., Lorincz A.T., Wacholder S., Sherman M., Scott D.R., Rush B.B., Glass A.G., Schiffman M. (2005). The elevated 10-year risk of cervical precancer and cancer in women with human papillomavirus (HPV) type 16 or 18 and the possible utility of type-specific HPV testing in clinical practice. J. Natl. Cancer Inst..

[17] Lowy D.R., Schiller J.T. (2006). Prophylactic human papillomavirus vaccines. J. Clin. Investig..

[18] Yang W., Mandal P.K., Liu Y. (2021). The Role of Oncogenes in the Development of HPV-Associated Cancers. Cancer Res..

[19] Villa L.L., Costa R.L.R., Petta C.A., Andrade R.P., Ault K.A., Giuliano A.R., Wheeler C.M., A Koutsky L., Malm C., Lehtinen M. (2005). Prophylactic quadrivalent human papillomavirus (types 6, 11, 16, and 18) L1 virus-like particle vaccine in young women: A randomised double-blind placebo-controlled multicentre phase II efficacy trial. Lancet Oncol..

[20] Palefsky J.M., Giuliano A.R., Goldstone S., Moreira E.D., Aranda C., Jessen H., Hillman R., Ferris D., Coutlee F., Stoler M.H. (2011). HPV vaccine against anal HPV infection and anal intraepithelial neoplasia. N. Engl. J. Med..

[21] Paavonen J., Jenkins D., Bosch F.X., Naud P., Salmerón J., Wheeler C.M., Chow S.-N., Apter D.L., Kitchener H.C., Castellsague X. (2007). Efficacy of a prophylactic adjuvanted bivalent L1 virus-like-particle vaccine against infection with human papillomavirus types 16 and 18 in young women: An interim analysis of a phase III double-blind, randomised controlled trial. Lancet.

[22] Lehtinen M., Paavonen J., Wheeler C.M., Jaisamrarn U., Garland S.M., Castellsagué X., Skinner S.R., Apter D., Naud P., Salmerón J. (2012). Overall efficacy of HPV-16/18 AS04-adjuvanted vaccine against grade 3 or greater cervical intraepithelial neoplasia: 4-year end-of-study analysis of the randomised, double-blind PATRICIA trial. Lancet Oncol..

[23] Franco E.L., Villa L.L., Sobrinho J.P., Prado J.M., Rousseau M.C., Desy M., Rohan T.E. (1999). Epidemiology of acquisition and clearance of cervical human papillomavirus infection in women from a high-risk area for cervical cancer. J. Infect. Dis..

[24] Castle P.E., Jeronimo J., Schiffman M., Herrero R. (2006). HPV testing in cervical cancer screening. Lancet.

[25] Schiffman M., Castle P.E., Jeronimo J., Rodriguez A.C., Wacholder S. (2007). Human papillomavirus and cervical cancer. Lancet.

[26] Muñoz N., Bosch F.X., de Sanjosé S., Herrero R., Castellsagué X., Shah K.V., Snijders P.J.F., Meijer C.J.L.M. (2003). Epidemiologic classification of human papillomavirus types associated with cervical cancer. N. Engl. J. Med..

[27] Franceschi S., Jaffe H. (2007). Cervical cancer screening of women living with HIV infection: A must in the era of antiretroviral therapy. Clin. Infect. Dis..

[28] Roberts S., Tindle R.W. (2022). Human Papillomavirus Vaccination and the Control of HPV-Associated Diseases. J. Infect. Diseases..

[29] Mantovani F., Banks L. (2001). The Human Papillomavirus E6 Protein and Its Contribution to Malignant Progression. Oncogene.

[30] McBride A.A. (2017). Oncogenic Human Papillomaviruses. Philos. Trans. R. Soc. B Biol. Sci..

[31] Harden M.E., Munger K. (2022). The Role of High-Risk Human Papillomavirus E6 Oncoproteins in Cancer. Pathogens.

[32] Longworth M.S., Laimins L.A. (2004). Pathogenesis of Human Papillomaviruses in Differentiating Epithelia. Microbiol. Mol. Biol. Rev..

[33] Narisawa-Saito M., Kiyono T. (2007). Basic Mechanisms of High-Risk Human Papillomavirus-Induced Carcinogenesis: Roles of E6 and E7 Proteins. Cancer Sci..

[34] Wallace N.A., Robinson K., Galloway D.A. (2017). Beta Human Papillomavirus E6 Expression Inhibits Stabilization of p53 and Increases Tolerance of Genomic Instability. J. Virol..

[35] Doorbar J., Quint W., Banks L., Bravo I.G., Stoler M., Broker T.R., Stanley M.A. (2012). The Biology and Life-Cycle of Human Papillomaviruses. Vaccine.

[36] Harwood C.A., Proby C.M. (2002). Human papillomaviruses and non-melanoma skin cancer. Curr. Opin. Infect. Dis..

[37] McLaughlin-Drubin M.E., Münger K. (2009). Oncogenic activities of human papillomaviruses. Virus Res..

[38] Cornet I., Gruffat H., Morin G., Banks L., Manet E., Calhoun E.S., Tommasino M. (2012). HPV16 infection and E6/E7-induced transformation of keratinocytes require the notch signaling pathway. Viruses.

[39] Iannacone M.R., Gheit T., Pfister H., Giuliano A.R., Messina J.L., Fenske N.A., Cherpelis B.S., Sondak V.K., Roetzheim R.G., Silling S. (2014). Case-Control Study of Genus-Beta Human Papillomaviruses in Plucked Eyebrow Hairs and Cutaneous Squamous Cell Carcinoma. Int. J. Cancer.

[40] Ramsay H.M., Reece S.M., Fryer A.A., Harden P.N. (2002). Clinical risk factors associated with nonmelanoma skin cancer in renal transplant recipients. Am. J. Kidney Dis..

[41] Ajila V., Shetty H., Babu S., Shetty V., Hegde S. (2015). Human Papilloma Virus Associated Squamous Cell Carcinoma of the Head and Neck. J Sex Transm Dis..

[42] Smith J. (2020). HPV and Skin Lesions: An Overview. Dermatol. Today.

[43] Brown A., Johnson P. (2019). Human Papillomavirus: Cutaneous Manifestations. J. Clin. Virol..

[44] White R., Green L. (2018). HPV-Associated Skin Conditions. Lancet Dermatol..

[45] Wright E. (2020). HPV Infection Mechanisms: An Overview. Curr. Dermatol. Rep..

[46] Anderson J. (2020). HPV-Related Cutaneous Lesions. Dermatol. Insights.

[47] Parker T., Davis L. (2019). HPV and Its Impact on Skin. JCAD.

[48] Humans I.W.G. (2007). Human papillomaviruses. IARC Monogr. Eval. Carcinog. Risks Hum..

[49] Arlette J.P., Trotter M.J. (2004). Squamous Cell Carcinoma In Situ of the Skin: History, Presentation, Biology and Treatment. Australas. J. Dermatol..

[50] Bailey R. (2019). HPV and Skin Pathology. J. Ski. Infect..

[51] Foster D. (2018). HPV and Skin Lesion Management. J. Dermatol. Ther..

[52] Nelson A., King P. (2017). HPV and Cutaneous Health. Dermatol. Insights Res..

[53] Butler R., Scott T. (2021). HPV-Associated Skin Lesions: Treatment Options. J. Dermatol..

[54] Evans L. (2020). HPV in Dermatologic Practice. J. Clin. Dermatol..

[55] Peterson J. (2019). HPV and Skin Disease: A Review. Dermatol. Rev..

[56] Frazer I.H. (2004). Development and Implementation of Prophylactic HPV Vaccines. Nat. Rev. Immunol..

[57] Kennedy E.M., Kornepati A.V., Goldstein M., Bogerd H.P., Poling B.C., Whisnant A.W., Chiang C.M., Cullen B.R. (2014). Inactivation of CRISPR-Cas9 Targets in HPV16- and HPV18-Infected Cells. J. Virol..

[58] Ferris R.L., Blumenschein G., Fayette J., Guigay J., Colevas A.D., Licitra L., Harrington K., Kasper S., Vokes E.E., Even C. (2016). Nivolumab for Recurrent Squamous-Cell Carcinoma of the Head and Neck. N. Engl. J. Med..

[59] Cohen E.E.W., Soulières D., Le Tourneau C., Dinis J., Licitra L., Ahn M.-J., Soria A., Machiels J.-P., Mach N., Mehra R. (2019). Pembrolizumab Versus Methotrexate, Docetaxel, or Cetuximab for Recurrent or Metastatic Head-and-Neck Squamous Cell Carcinoma (KEYNOTE-040): A Randomised, Open-Label, Phase 3 Study. Lancet.

[60] Bommareddy P.K., Shettigar M., Kaufman H.L. (2018). Integrating Oncolytic Viruses in Combination Cancer Immunotherapy. Nat. Rev. Immunol..

[61] Musial C., Kuban-Jankowska A., Gorska-Ponikowska M. (2020). Beneficial Properties of Green Tea Catechins. Int. J. Mol. Sci..

[62] Zhong Z., McCafferty D.G., Inturi S., Zhou S., Wang L., Ohlsen C.A., DiMeo A., Rodriguez R., Zou Z. (2019). Nanotechnology-Based Therapeutics for HPV-Related Cancers. Nanomedicine.

[63] Egger G., Liang G., Aparicio A., Jones P.A. (2004). Epigenetics in Human Disease and Prospects for Epigenetic Therapy. Nature.

[64] Cubie H.A. (2013). Diseases Associated with Human Papillomavirus Infection. Virology.

[65] Akgül B., Cooke J.C., Storey A. (2005). HPV-Associated Skin Disease. J. Pathol..

[66] Julius J.M., Ramondeta L., Tipton K.A., Lal L.S., Schneider K., Smith J.A. (2012). Clinical Perspectives on the Role of the Human Papillomavirus Vaccine in the Prevention of Cancer. Pharmacotherapy.

[67] Shimizu A., Yamaguchi R., Kuriyama Y. (2023). Recent Advances in Cutaneous HPV Infection. J. Dermatol..

[68] Handisurya A., Schellenbacher C., Kirnbauer R. (2009). Diseases Caused by Human Papillomaviruses (HPV). JDDG J. Dtsch. Dermatol. Ges..

[69] Neagu N., Dianzani C., Venuti A., Bonin S., Voidăzan S., Zalaudek I., Conforti C. (2022). The Role of HPV in Keratinocyte Skin Cancer Development: A Systematic Review. J. Eur. Acad. Dermatol. Venereol..

[70] Brüggmann D., Kayser L., Jaque J., Bundschuh M., Klingelhöfer D., Groneberg D.A. (2018). Human papilloma virus: Global research architecture assessed by density-equalizing mapping. Oncotarget.

[71] Campos-Romero A., Anderson K.S., Longatto-Filho A., Esparza M.A.L.-R., Morán-Portela D.J., Castro-Menéndez J.A., Moreno-Camacho J.L., Calva-Espinos D.Y., Acosta-Alfaro M.A., Meynard-Mejía F.A. (2019). The burden of 14 hr-HPV genotypes in women attending routine cervical cancer screening in 20 states of Mexico: A cross-sectional study. Sci. Rep..

[72] de Sanjosé S., Brotons M., Pavón M.A. (2018). The natural history of human papillomavirus infection. Best Pract. Res. Clin. Obstet. Gynaecol..

[73] Faridi R., Zahra A., Khan K., Idrees M. (2011). Oncogenic potential of Human Papillomavirus (HPV) and its relation with cervical cancer. Virol. J..

[74] Forman D., de Martel C., Lacey C.J., Soerjomataram I., Lortet-Tieulent J., Bruni L., Vignat J., Ferlay J., Bray F., Plummer M. (2012). Global burden of human papillomavirus and related diseases. Vaccine.

[75] Vashisht S., Mishra H., Mishra P.K., Ekielski A., Talegaonkar S. (2019). Structure, genome, infection cycle and clinical manifestations associated with human papillomavirus. Curr. Pharm. Biotechnol..

[76] Yu L., Majerciak V., Zheng Z.M. (2022). HPV16 and HPV18 genome structure, expression, and post-transcriptional regulation. Int. J. Mol. Sci..

[77] Graham S.V. (2017). The human papillomavirus replication cycle, and its links to cancer progression: A comprehensive review. Clin. Sci..

[78] Regauer S., Reich O. (2021). The origin of Human Papillomavirus (HPV)-induced cervical squamous cancer. Curr. Opin. Virol..

[79] Loopik D.L., Bentley H.A., Eijgenraam M.N., IntHout J., Bekkers R.L.M., Bentley J.R. (2021). The natural history of cervical intraepithelial neoplasia grades 1, 2, and 3: A systematic review and meta-analysis. J. Low. Genit. Tract. Dis..

[80] Srisuttayasathien M., Manchana T. (2021). Adherence to follow-up in women with cervical intraepithelial neoplasia grade 1. Taiwan J. Obstet. Gynecol..

[81] Li T.Y., Wu Z.N., Jiang M.Y., Cui J.F., Liu B., Chen F., Chen W. (2018). Association between high-risk human papillomavirus DNA load and cervical lesions in different infection status. Zhonghua Zhong Liu Za Zhi.

[82] Kamal M. (2022). Cervical pre-cancers: Biopsy and immunohistochemistry. CytoJournal.

[83] Nunes R.A.L., Morale M.G., Silva G.Á.F., Villa L.L., Termini L. (2018). Innate immunity and HPV: Friends or foes. Clinics.

[84] Sharifian K., Shoja Z., Jalilvand S. (2023). The interplay between human papillomavirus and vaginal microbiota in cervical cancer development. Virol. J..

[85] Okunade K.S. (2020). Human papillomavirus and cervical cancer. J. Obstet. Gynaecol..

[86] Kang S.D., Chatterjee S., Alam S., Salzberg A.C., Milici J., van der Burg S.H., Meyers C. (2018). Effect of productive human papillomavirus 16 infection on global gene expression in cervical epithelium. J. Virol..

[87] St Laurent J., Luckett R., Feldman S. (2018). HPV vaccination and the effects on rates of HPV-related cancers. Curr. Probl. Cancer.

[88] Paller A., Jaworski J.C., Simpson E.L., Boguniewicz M., Russell J.J., Block J.K., Tofte S., Dunn J.D., Feldman S.R., Clark A.R. (2018). Major comorbidities of atopic dermatitis: Beyond allergic disorders. Am. J. Clin. Dermatol..

[89] Ezeonwumelu I.J., Garcia-Vidal E., Ballana E. (2021). JAK-STAT Pathway: A novel target to tackle viral infections. Viruses.

[90] Kim B.S., Howell M.D. (2021). The pathophysiology of atopic dermatitis. Clin. Rev. Allergy Immunol..

[91] Bhatia R., Kavanagh K., Cubie H.A., Serrano I., Wennington H., Hopkins M., Pan J., Pollock K.G., Palmer T.J., Cuschieri K. (2016). Use of HPV Testing for Cervical Screening in Vaccinated Women—Insights from the SHEVa (Scottish HPV Prevalence in Vaccinated Women) Study. Int. J. Cancer.

[92] Hasche D., Akgül B. (2023). Prevention and Treatment of HPV-Induced Skin Tumors. Cancers.

[93] Bouwes Bavinck J.N., Feltkamp M.C.W., Green A.C., Fiocco M., Euvrard S., Harwood C.A., Nasir S., Thomson J., Proby C.M., Naldi L. (2018). Human papillomavirus and posttransplantation cutaneous squamous cell carcinoma: A multicenter, prospective cohort study. Am. J. Transplant..

[94] Mlynarczyk-Bonikowska B., Rudnicka L. (2024). HPV Infections—Classification, Pathogenesis, and Potential New Therapies. Int. J. Mol. Sci..

[95] Tommasino M. (2019). HPV and skin carcinogenesis. Papillomavirus Res..

[96] Que S.K.T., Zwald F.O., Schmults C.D. (2018). Cutaneous squamous cell carcinoma: Incidence, risk factors, diagnosis, and staging. J. Am. Acad. Dermatol..

[97] Spurgeon M.E., Lambert P.F. (2020). Mus musculus Papillomavirus 1: A New Frontier in Animal Models of Papillomavirus Pathogenesis. J. Virol..

[98] Antonsson A., Erfurt C., Hazard K., Holmgren V., Simon M., Kataoka A., Hossain S., Hakangard C., Hansson B.G. (2003). Prevalence and type spectrum of human papillomaviruses in healthy skin samples collected in three continents. J. Gen. Virol..

[99] Gay J., Johnson N., Kavuru V., Phillips M. (2021). Utility of the Human Papillomavirus Vaccination in Management of HPV-associated Cutaneous Lesions. Ski. Ther. Lett..

[100] Tschandl P., Rosendahl C., Kittler H. (2014). Cutaneous Human Papillomavirus Infection: Manifestations and Diagnosis. Hum. Papillomavirus.

[101] Yassin A., Dixon D.R., Oda D., London R.M. (2016). Diagnosis and Clinical Management of Human Papilloma Virus–Related Gingival Squamous Cell Carcinoma in a Patient With Leukemia: A Case Report. Clin. Adv. Periodontics.

[102] Schiller J.T., Lowy D.R. (2012). Understanding and Learning from the Success of Prophylactic Human Papillomavirus Vaccines. Nat. Rev. Microbiol..

